# Antimicrobial activity and the presence of virulence factors and bacteriocin structural genes in *Enterococcus faecium* CM33 isolated from ewe colostrum

**DOI:** 10.3389/fmicb.2015.00782

**Published:** 2015-07-29

**Authors:** Yousef Nami, Babak Haghshenas, Minoo Haghshenas, Ahmad Yari Khosroushahi

**Affiliations:** ^1^Institute of Biosciences, University Putra MalaysiaSelangor, Malaysia; ^2^School of Medicine, Shahid Beheshti University of Medical SciencesTehran, Iran; ^3^Drug Applied Research Center, Tabriz University of Medical SciencesTabriz, Iran; ^4^Department of Pharmacognosy, Faculty of Pharmacy, Tabriz University of Medical SciencesTabriz, Iran

**Keywords:** antimicrobial activity, *Enterococcus faecium*, enterocins, probiotic, virulence genes

## Abstract

Screening of lactic acid bacteria (LAB) isolated from ewe colostrum led to the identification and isolation of *Enterococcus faecium* CM33 with interesting features like high survival rates under acidic or bile salts condition, high tolerance for the simulated gastrointestinal condition, and high adhesive potential to Caco-2 cells. According the inhibition of pathogen adhesion test results, this strain can reduce more than 50% adhesion capacity of *Escherichia coli, Shigella flexneri, Klebsiella pneumoniae, Listeria monocytogenes*, and *Staphylococcus aureus* to Caco-2 cells. Based on the antibiotic sensitivity test findings, *E. faecium* CM33 was susceptible to gentamycin, vancomycin, erythromycin, ampicillin, penicillin, tetracycline, and rifampicin, but resistant to chloramphenicol, clindamycin, and kanamycin. Upon assessment of the virulence determinants for *E. faecium* CM33, this strain was negative for all tested virulence genes. Furthermore, the genome of this strain was evaluated for the incidence of the known enterocin genes by specific PCR amplification and discovered the genes encoding enterocins A, 31, X, and Q. Based on this study findings, the strain *E. faecium* CM33 can be considered as a valuable nutraceutical and can be introduced as a new potential probiotic.

## Introduction

The health promoting effects of probiotic bacteria are strain-specific, hence their discrimination and identification is very important and crucial under strain level by applying an efficient and valid strain-identification techniques such as 16S rRNA sequencing. Besides, some identification methods can be utilized for assessment on the safety aspect and ecological properties of probiotics (Pereira and Gibson, 2002; Senok, 2009). The choice of appropriate probiotics is the most important base for enhancing the bio-therapeutic achievement in nutraceutical/pharmaceutical products. Several important characters of bacteria used as probiotics are the ability to survive in gastric conditions and adhere to the host intestinal cells (Kirjavainen et al., 1998; Dunne et al., 2001). However, *in vitro* procedures for choice of promising probiotics are necessary because *in vivo* methods are costly and need agreement by ethical committees.

Caco-2 cells have been effectively applied to find the mechanism of cellular adhesion of non-pathogenic lactic acid bacteria (LAB; Tuomola and Salminen, 1998). It has also been explained that enterococci produce antimicrobial substances such as bacteriocins, hydrogen peroxide, and lactic acid (Sparo et al., 2006; Franz et al., 2007), which could elucidate their ability against several pathogens such as *Staphylococcus aureus* and *Listeria monocytogenes*. The connection of *Enterococcus* bacteria with human diseases has raised concern regarding their use as probiotic microorganisms (Franz et al., 2011). Several enterococcal strains are able to have antiviral activity (Wang et al., 2013), beneficial effects in anti-tumoral protective responses (Thirabunyanon and Hongwittayakorn, 2013) and can restore the microbiota balance in antibiotic-induced dysbiosis (Tarasova et al., 2010). *Enterococcus* bacteria, utilized in fermentation of vegetable products, meat, and dairy, are the reason of clinical infections. Therefore, it is vital to evaluate the virulence genes as well the antibiotic resistance profile.

Although the safety aspect of this genus is still blurred, members of enterococci are now engaged as probiotic bacteria to improve the immunity and human health (Brandão et al., 2010). The capability of these bacteria to make enterocins is remarkable and can be applied as food bio-preservatives (Khan et al., 2010). Bacteriocins are small, ribosomally synthesized and heat-stable peptides that apply antimicrobial activity against pathogenic bacteria or food spoilage, including *Staphylococcus aureus, Clostridium* spp. *Bacillus* spp., *L. monocytogenes*, and *Campylobacter* spp. (Nes et al., 2007; Line et al., 2008). Franz et al. (2007) categorized bacteriocins produced by *Enterococcus* bacteria into four different classes: Class I is lantibiotic enterocins such as cytolysin (CylLL/S; Gilmore et al., 1994); Class II is small, non-lantibiotic enterocins; Class III is cyclic enterocins for instance enterocin AS-48 (Eijsink et al., 2002); and Class IV is large proteins like enterolysin A (Hickey et al., 2003). There are three subclasses within class II including subclass IIa such as enterocin A and P (Ent A and Ent P), which are pediocin-like bacteriocins; subclass IIb like enterocin L50 and Q (Ent L50A/B and Ent Q), which required two peptides for full antimicrobial activity and subclass IIc like enterocin B (Ent B), which are other linear and non-pediocin-type enterocins (Casaus et al., 1997).

Although there are rising concern about the safety of *Enterococcus* bacteria because of their relationship with several human infections (Brandão et al., 2010), a lot of works specify that enterococci play a key function in the improvement of the sensory properties of fermented foods like olives, sausages and cheese (Moreno et al., 2006). Carlos et al. (2010) elucidated a lot of virulence factors and their actions in *Enterococcus* bacteria (Carlos et al., 2010). These include the *cylA* which is in charge of the transportation and activation of the cytolisin; *cylB* and *cylM* involved in the post-translational modification; *esp* gene in charge of a cell wall protein involved in immune evasion; *agg* encodes an aggregation protein which is responsible for adherence to eukaryotic cells; *gelE* is in charge of toxin production that hydrolyzes gelatine and other compounds, and finally *cpd, ccf*, and *cad* genes encode sex pheromones, which are in charge of facilitating conjugation.

Because of the high potential of *Enterococcus* bacteria in health and food, the purpose of current study was to find out the antimicrobial activity and the presence of bacteriocin structural genes and virulence factors in *Enterococcus faecium* CM33 isolated from ewe colostrum to assess the potential probiotic properties and safety aspect of use of this strain.

## Materials and Methods

### Bacteria Isolation

*Enterococcus faecium* CM33 was isolated from ewe colostrum by culturing in de Man Rogosa and Sharpe broth (MRS) and following incubation of 50 μl of cultured medium onto MRS agar supplemented with 6.5% (w/v) NaCl (MRS-NaCl) for 18 h at 37°C in aerobic conditions. After enrichment, five passages by isolated colony, the pure bacterium isolate was obtained.

### PCR Amplification of Isolate

The amplification of 16S rRNA gene (1500 bp) of the isolate was performed by the primers (F 5′-AGAGTTTGATC CTGGCTCAG-3′ and R 5′-GGCTGCTGGCACGTAGTTAG-3′), which those primarily used by Kullen et al. (2000). The amplification reaction was performed in a thermocycler (Thermo Electron Corporation, Waltham, MA, USA) with the following thermocycle program: Four min at 96°C as an initial denaturation; 30 s at 96°C as a denaturation for 30 cycles; 30 s at 48°C as an annealing and 45 s at 72 C as an extension, with a final extension step for 4 min at 72°C. The total volume of reaction was 50 μl. The PCR product was visualized through 0.8% (w/v) agarose gel (Sigma Chemical Co., Poole, UK) electrophoresis by using ethidium bromide staining (Kullen et al., 2000). PCR product was sequenced by the Macrogen DNA Sequencing Service (Korea). The sequence was interrogated through the BLAST search of the NCBI database. The isolate was identified on account of the highest matching score.

### Effects of Heat, pH, and Hydrolytic Enzymes

This strain was cultured in MRS broth for 18 h at 37°C. After 10 min centrifugation at 12000 g, cells were removed and the cell free supernatant (metabolites) was utilized for additional characterization. To determine the effect of pH on the metabolites action, pH of the crude supernatant was adjusted in the range between 1.5 and 9 by adding sterile 1 M HCl or 1 M NaOH. Sample was incubated at room temperature for 4 h. Thermostability of metabolites was determined at diverse temperatures. Crude supernatant was autoclaved at 121°C for 15 min and then incubated at different temperatures (4, -20, and -70C) for 24 h, 48 h, 2 weeks, and 1 month.

The sensitivity of cell free supernatant toward different proteolytic enzymes, amylase, and catalase was investigated. One mg/ml of enzymes including proteinase K, pronase E, trypsin, and chymotrypsin were prepared in 15 mM sodium phosphate buffer (pH 7.2) and pepsin was prepared in glycine buffer pH 2. Before incubating for 2 h at 37°C, proteases at a final concentration of 0.1 mg/ml were added. The crude cell free supernatant was treated with catalase at 0.1 mg/ml to remove potential inhibitory effects because of production of hydrogen peroxide.

### Targeting Bacteriocin Genes

To detect genes encoding the known enterocins, genomic DNA of strain CM33 was used as template. PCR were performed in a Hybrid PCR Sprint thermocycler (Thermo Electron Corporation, Waltham, MA, USA) in 20 ml reaction mixture (10 pmoles of each primer, 1 mL DNA template, and 1.25 U Taq polymerase). The PCR conditions for enterocin-encoded genes are illustrated in **Table 1**. The PCR products were visualized by electrophoresis in 1.5% agarose gels.

**Table 1:** Primer sequences for PCR amplification of enterocin genes in *Enterococcus faecium* CM33.

Genes	Primers	Sequences 5′→3′	Size (bp)	Tm (°C)	PCR amplification	Reference
A B P X 31 Q AS48 *Cyl* L50A	EntA-F EntA-R EntB-F EntB-R P-F P-R EntX-F EntX-R Ent31-F Ent31-R EntQ-F EntQ-R EntAS48-F EntAS48-R Cyl-F Cyl-R EntL50-F EntL50-R	AAATATTATGGAAATGGAGTGTAT CTCGTTAAGGTCCCTTCACG CAAAATGTAAAAGAATTAAGTACG AGAGTATACATTTGCTAACCC GCTACGCGTTCATATGGTAATGGT ATGTCCCATACCTGCCAAACCAGAAGC GTTTCTGTAAAAGAGATGAAAC CATACCAATTACTAATTCTCC CCTACGTATTACGGAAATGGT GCCATGTTGTACCCAACCATT CAAGAAATTTTTTCCCATGGC ACGCTATGGTAAAAATTCTTC GAGGAGTTTCATGATTTAAAG CGAACCATTAAATTGTTATAC GGCGGTATTTTTACTGGAGTN CCTACTCCTAAGCCTATGGTA GGAGCAATCGCAAAATTAG TAACCTCTTCCTACCCGTTA	475 201 132 500 130 95 185 248 150	56 56 60 50 58 55 50 58 55	– – – + + + – + –	Du Toit et al. (2000) De Vuyst et al. (2003) Cintas et al. (2000) Edalatian et al. (2012) De Vuyst et al. (2003) Citti et al. (2002) Du Toit et al. (2000) De Vuyst et al. (2003) Du Toit et al. (2000)

### Bile Tolerance Test

The method previously described by Pereira and Gibson (2002) was used to measure the viability of strain in bile salt. The isolate was grown in MRS medium supplemented with 0.5% (w/v) oxgall then was incubated for 4 h at 37°C. To determine of cell count, aliquots were plated onto MRS-agar medium at times 0, 1, 3, and 4 h. Control tube was without bile salt (Pereira and Gibson, 2002).

### Survivability in Simulated *In Vitro* Digestion

Simulated *in vitro* digestion was carried out to evaluate the survival rate of strain CM33 in similar condition of the human gastrointestinal tract. To simulate the stomach digestion, samples (pH 3.0) were treated with 5% (w/v) pepsin (Sigma Chemical Co., Poole, UK) then were incubated for 120 min at 37°C with gentle agitation. The intestinal digestion was simulated by inoculating solutions of 0.1% (w/v) pancreatin (Sigma Chemical Co., Poole, UK) and 0.3% (w/v) bile oxgall. The samples were incubated for 180 min at 37°C with gentle shaking. Samples were removed before and after gastric and intestinal digestion to measure cell count. The serially diluted aliquots were cultured on MRS agar medium and were incubated for 24 h under anaerobic conditions (Seiquer et al., 2001).

### Adhesion Ability to Caco-2 Cells

Caco-2, the colon adenocarcinoma cell line, was used to evaluate the adhesion ability of *E. faecium* CM33 to human epithelial cells. RPMI medium supplemented with 10% (v/v) fetal bovine serum and 1% (v/v) penicillin/streptomycin mixture (Sigma Chemical Co., Poole, UK) was used to culture human colon cancer cells. Cells were seeded on 24-well tissue culture plates and incubated at 37°C in 5% CO_2_ in a humidified atmosphere. Before the adhesion assay, fresh antibiotic-free RPMI was used to wash wells, which have a monolayer of Caco-2 cells. Then, each well was inoculated with 1 × 10^7^ cfu/ml of *E. faecium* in a total volume of 1 ml and incubated at 37°C for 3 h in an atmosphere of 5% (v/v) CO_2_. The wells were washed thrice with a pre-warmed phosphate buffer saline (Sigma Chemical Co., Poole, UK) to remove non-attached cells of *E. faecium*. To detach the cells, 1 ml of 1% Triton X-100 (Sigma Chemical Co., Poole, UK) was added to each well with gently stirring for 5–10 min and then the viable human colon cancer cells, Caco-2, were counted. Finally, bacteria and Caco-2 cells were cultured onto MRS agar by the pure plate method and incubated at 37°C anaerobically. The total number of bacteria attached to viable Caco-2 cells expressed as bacterial adhesion and carried out in triplicate.

### Inhibition of Pathogen Adhesion to Caco-2 cells

Caco-2, human colon cells, were used to assess the effect of *E. faecium* treatment on the pathogen interaction with cells. *E. faecium* and pathogens (*Escherichia coli, S. flexneri, K. pneumonia*, and *S. aureus*) were cultured in MRS and LB, respectively. Overnight culture of *E. faecium* and pathogens were harvested and washed thrice with phosphate buffer saline. Bacteria were diluted in antibiotic-free RPMI and 0.5 ml/well (10^8^ CFU per well) of *E. faecium* suspension was inoculated to 24-well tissue culture plates. To evaluate the capability of *E. faecium* to restrain pathogenic bacteria adhesion to Caco-2 cells, simultaneous addition of 10^8^ CFU per well of tested pathogens was performed separately. Plates were incubated 1 h at 37°C and washed twice with sterile PBS then 1% (v/v) Triton X-100 (Sigma Chemical Co., Poole, UK) in deionized water for 5 min was used to lyse the cell-associated pathogens. To find out the number of viable cell-linked bacteria by colony forming counts, the suitable dilutions of the lysate were placed on LB agar. The inhibition analysis was calculated in three independent trials and each experiment was carried out in triplicate.

### Antimicrobial Activity

To determine and identify inhibitory substances of metabolites produced by *E. faecium* CM33, well-diffusion method was performed (Hechard et al., 1990). Overnight cultures of the indicator strains were cultured in MRS agar at 37°C for 18 h. 50 μl of filtered crude supernatant of CM33 were dropped to each well (5 mm) into agar plates. The suspensions were inoculated to the wells and permitted to diffuse for 4 h at room temperature into MRS agar. The inhibition zone produced around the wells was determined after 24 h incubation at the optimum growth temperature of the indicator strains. The diameter of the produced inhibition zones around the wells was determined by digital caliper. These diameters were classified to four different groups including no inhibition (–), weak (+), good (++), and strong (+++). All trials were carried out in triplicate.

### PCR Detection of Virulence Factors in Enterococci

Detection of genes encoding potential virulence factors in *E. faecium* CM33 was performed by PCR. Total bacterial DNA used as template in PCR reactions was isolated by the alkaline lysis method (Baele et al., 2001). Virulence genes evaluated in this study include: cytolysin (CylA, CylB, CylM; Semedo et al., 2003; Vankerckhoven et al., 2004), enterococcal surface protein (esp; Vankerckhoven et al., 2004), collagen-binding protein (ace; Omar et al., 2004), gelatinase (gelE), aggregation substance (agg; Eaton and Gasson, 2001; Ahmed et al., 2012), sex pheromone peptides (cpd; Abriouel et al., 2008), cell-wall anchored collagen adhesion (acm; Cariolato et al., 2008; Nallapareddy et al., 2008), and van A, van B, van C2 (Satake et al., 1997). *E. faecium* ATCC 51299 and ATCC 29212 were controls for this test. PCR was used to detect of genes encoding these factors using several primers (**Table 2**) which have been described by Eaton and Gasson (2001).

**Table 2:** Primers used for screening of virulence factors genes.

Gene	Primer	Sequence 5′→3′	Product size (bp)	Reference
*CylA*	cytI cytIIb	ACTCGGGGATTGATAGGC GCTGCTAAAGCTGCGCTT	688	Vankerckhoven et al. (2004)
*CylB*	cytB1 cytB2	AAGTACACTAGTAGAACTAAGGGA ACAGTGAACGATATAACTCGCTATT	2020	Semedo et al. (2003)
*CylM*	cylM1 cylM2	AAAAGGAGTGCTTACATGGAAGAT CATAACCCACACCACTGATTCC	2940	Semedo et al. (2003)
*esp*	esp14F esp12R	AGATTTCATCTTTGATTCTTGG AATTGATTCTTTAGCATCTGG	510	Vankerckhoven et al. (2004)
*ace*	ace-F ace-R	GAATTGAGCAAAAGTTCAATCG GTCTGTCTTTTCACTTGTTTC	1008	Ben Omar et al. (2008)
*gelE*	gelE-F gelE-R	ACCCCGTATCATTGGTTT ACGCATTGCTTTTCCATC	419	Reviriego et al. (2005)
*agg*	agg-F agg-R	AAGAAAAAGAAGTAGACCAAC AAACGGCAAGACAAGTAAATA	1553	Eaton and Gasson (2001)
*cpd*	cpd-F cpd-R	TGGTGGGTTATTTTTCAATTC TACGGCTCTGGCTTACTA	782	Reviriego et al. (2005)
*van A*	van *C_2_-*1 van *C_2_-*2	CGGGGAAGATGGCAGTAT CGCAGGGACGGTGATTTT	732	Satake et al. (1997)
*van B*	van *C_2_-*1 van *C_2_-*2	CGGGGAAGATGGCAGTAT CGCAGGGACGGTGATTTT	635	Satake et al. (1997)
*van C_2_*	van *C_2_-*1 van *C_2_-*2	CGGGGAAGATGGCAGTAT CGCAGGGACGGTGATTTT	484	Satake et al. (1997)

### Interpretation of Results

Data were analyzed using SPSS 19.0 software (SPSS Inc., an IBM Company, Schaumburg, IL, USA). One-way ANOVA followed by multiple mean comparisons Duncan’s test were used to assess statistical differences in multiple groups. Values are mean ± SE and *p* ≤ 0.05 is considered statistically significant.

## Results

### Bacteria Isolation

Screening of different dairy products like colostrum, yogurt, curd, cheese, and yogurt drink led to the isolation of *E. faecium* CM33 from ewe colostrum. After DNA extraction, the 16S rRNA fragments were amplified by PCR. According to 16S rRNA identification, 50 of the isolated bacteria were classified into three major groups of LAB, namely, 14% enterococci, 66% lactobacilli, and 20% lactococci. The isolates classified as LAB were also separated and identified by sequencing. After sequencing, the *Enterococcus* strains were categorized into *E. lactis, E. hirae, E. avium, E. durans, E. faecalis*, and *E. faecium*. The *Lactobacillus* strains representing 66% were classified into *L. acidophilus* and *L. plantarum*. The *Lactococcus* species were classified only into one species (*Lactococcus lactis*). Probiotic characterization of these isolates showed that *E. faecium* CM33 has the best properties such as tolerance to acid and bile, antimicrobial activity and antibiotic resistance thus it was selected for further analysis.

### Effects of pH, Heat, and Hydrolytic Enzymes on *E. faecium* Bacteriocin

The stability and activity of *E. faecium* CM33 bacteriocins has been shown in **Table 3**. Bacteriocins were active over broad ranges of pH from 1.5 to 9. It was also stable after autoclaving at 121°C for 15 min. The treatment of cell free supernatant of *E. faecium* CM33 with amylase, catalase, and proteolytic enzymes showed that amylase and catalase enzymes do not influence on the bacteriocin activity, while proteolysis enzymes affect on the bacteriocin activity after 2 h.

**Table 3:** Effects of pH, heat, and enzymes (concentration 1 mg/ml) on the antibacterial activity present in *E. faecium* CM33 culture metabolites.

Treatments	Antibacterial activity
**pH** 1.5–9 **Heat** 121° C for 15 min **Enzymes** Pepsin Trypsin Chymotrypsin Proteinase K Pronase E Catalase Amylase	+ + – – – + + – –

### PCR Analysis of Bacteriocin Genes

PCR amplification using specific primers as shown in **Table 1** was used to screen DNA of *E. faecium* CM33 for the existence of the known enterocin genes. This study focused on enterocin genes including *Cyl*, A, P, 31, Q, X, L50A, and AS-48. *E. faecium* CM33 showed four bands: 95, 130, 248, and 500 bp corresponding to the specific amplification of enterocins Q, 31, *Cyl*, and X, respectively.

### Survivability in Bile Salt and Simulated Digestion

*Enterococcus faecium* CM33 was incubated in 0.3% bile salts for 4 h. It showed the survival rate of >93% after this incubation period. One of the most desirable features needed for probiotics is the ability to survive in the GI tract. For further analysis, *E. faecium* CM33 was evaluated using simulated digestion test. This test revealed that this strain had 57.6% digestion survivability.

### Adhesion Assay to Caco-2 Cells

The strain was tested further for its capability to colonize Caco-2 cells. *E. faecium* CM33 showed the acceptable adhesion ability with an adhesion value of 32 × 10^4^ cfu/ml. *E. faecium* ATCC 29212, which used as a control to compare their adhesion ability, could not significantly adhere to Caco-2 cells.

### Inhibition of Pathogens Adhesion

In the presence of *E. faecium* CM33, the inhibition of pathogens adhesion was presented in **Table 4**. All the adherent pathogens were clearly reduced by co-culture with *E. faecium*. The reduction of *E. coli, S. flexneri, K. pneumoniae*, and *S. aureus* adhesion to Caco-2 cells was more than 50% added with *E. faecium* 10^8^ CFU per well.

**Table 4:** Inhibition of pathogens adhesion by co-culture with *E. faecium* CM33.

Pathogens	Adhesive pathogen count (10^5^ CFU ml^-1^)	
	Untreated	Co-cultured
*Clostridium difficile* *Clostridium histolyticum* *Staphylococcus aureus* *Staphylococcus saprophyticus* *Salmonella typhimurium* *Candida albicans* *Escherichia coli 0157* *Bacillus cereus* *Listeria monocytogenes* *Klebsiella pneumoniae* *Shigella flexneri* *Pseudomonas aeroginosa* *Streptococcus mutans*	5.3 ± 0.4 3.8 ± 0.2 3.4 ± 0.3 4.6 ± 0.2 7.1 ± 0.3 6.4 ± 0.4 8.5 ± 0.2 6.6 ± 0.3 5.4 ± 0.2 8.3 ± 0.4 4.9 ± 0.3 5.2 ± 0.2 4.4 ± 0.4	3.7 ± 0.3 2.8 ± 0.4 1.1 ± 0.2 3.2 ± 0.3 4.8 ± 0.4 3.6 ± 0.3 3.8 ± 0.2 4.0 ± 0.4 2.5 ± 0.2 4.1 ± 0.3 1.3 ± 0.4 3.7 ± 0.3 2.7 ± 0.2

*Values are mean ± SE.*

### Antimicrobial Activity

The strain CM33 isolated from colostrum displayed antimicrobial activity against all the tested indicator microorganisms (**Table 5**). The most sensitive indicators were *L. monocytogenes*, followed by *Candida albicans*. On the contrary, *Clostridium difficile, Clostridium histolyticum, Salmonella typhimurium*, and *Streptococcus mutans* were the less sensitive indicator microorganisms.

**Table 5:** The antimicrobial activity of *E. faecium* CM33 against pathogens.

Pathogenic microorganisms	Source	Supernatant
*Clostridium difficile* *Clostridium histolyticum* *Staphylococcus aureus* *Staphylococcus saprophyticus* *Salmonella typhimurium* *Candida albicans* *Escherichia coli 0157* *Bacillus cereus* *Listeria monocytogenes* *Klebsiella pneumoniae* *Shigella flexneri* *Pseudomonas aeroginosa* *Streptococcus mutans*	ATCC 43255 ATCC 19401 ATCC 25923 PTCC 1440 ATCC 14028 ATCC 10231 PTCC 1276 ATCC 11778 PTCC 1163 ATCC 10031 PTCC 1234 PTCC 1181 ATCC 35668	+ + ++ ++ + +++ ++ ++ +++ ++ ++ ++ +

### Virulence Genes

The presence of virulence genes in genomic DNA of *E. faecium* CM33 was screened by PCR. Currently several enterococci are being used as probiotics but they should be carefully assessed to ensure that they are not pathogenic. *E. faecium* CM33 was found to be negative for all tested virulent genes (**Table 6**). In the case of van A, van B and van C_2_ genes, it is essential to note that this result associate with the lack of resistance to vancomycin.

**Table 6:** Detection of virulence-related genes in enterococcal strains used in this research.

Strain	Species	Virulent genes
ATCC 29212 ATCC 51299 CM33	*E. faecium* *E. faecium* *E. faecium*	*gelE, cylA* *gelE, vanB, ccf, agg* None

## Discussion

The food industry is requested to present increasingly functional foods containing beneficial components because of the great interest on health-oriented nutritional habits. Bacteria generally recognize as probiotic commonly belong to LAB. These microorganisms, as commensals in the human gastrointestinal tract, show a long history of use in foods and fermented products (Haghshenas et al., 2014a,b, 2015; Nami et al., 2014a,b,c). *Enterococcus* bacteria have various helpful functions in the dairy industry (Haghshenas et al., 2014c; Nami et al., 2014d). As starters, enterococci fulfill a considerable function in developing flavor progress and quality of cheeses. As probiotics, LAB are able to be used for healing of gut disorders in both humans and animals since these bacteria contribute to the intestinal health of the host by the development of gut microbial balance (Rehaiem et al., 2014). On the other hand, some requirements should be fulfilled to introduce a certain strain as a probiotic (Collins et al., 1998) and probiotic activities should be confirmed in well-made human studies. The aim of this paper was to evaluate safety and the potential beneficial properties of *E. faecium* CM33 isolated from ewe colostrum.

The most important characteristics required for microorganisms to be probiotics is their ability to survive while passing through the upper digestive tract to reach the large intestine, where their positive functions are predictable (Bezkorovainy, 2001; Tuomola et al., 2001; Nueno-Palop and Narbad, 2011). Probiotics can adhere to epithelial cells and should tolerate high acidic pH and high bile salt conditions in the gastric and intestinal fluid. Resistance to acid is of the popular properties used during the screening of potential probiotic strains. Another important mechanism in resisting intestinal pathogens and preventing diseases is the competition of probiotic bacteria with pathogens to adhere and colonize (Pascual et al., 2008). In this study, *E. faecium* CM33 isolated from ewe coloctrum dairy products was capable to survive both in bile salts and the gastric simulated digestion condition. This strain was further evaluated for their adhesion ability to Caco-2 cells. Data showed that *E. faecium* CM33 possessed an ability to resist the digestion conditions and was able to adhere to Caco-2 cells. These results show that *E. faecium* CM33 could be a suitable candidate to be as potential new probiotic.

**FIGURE 1:**
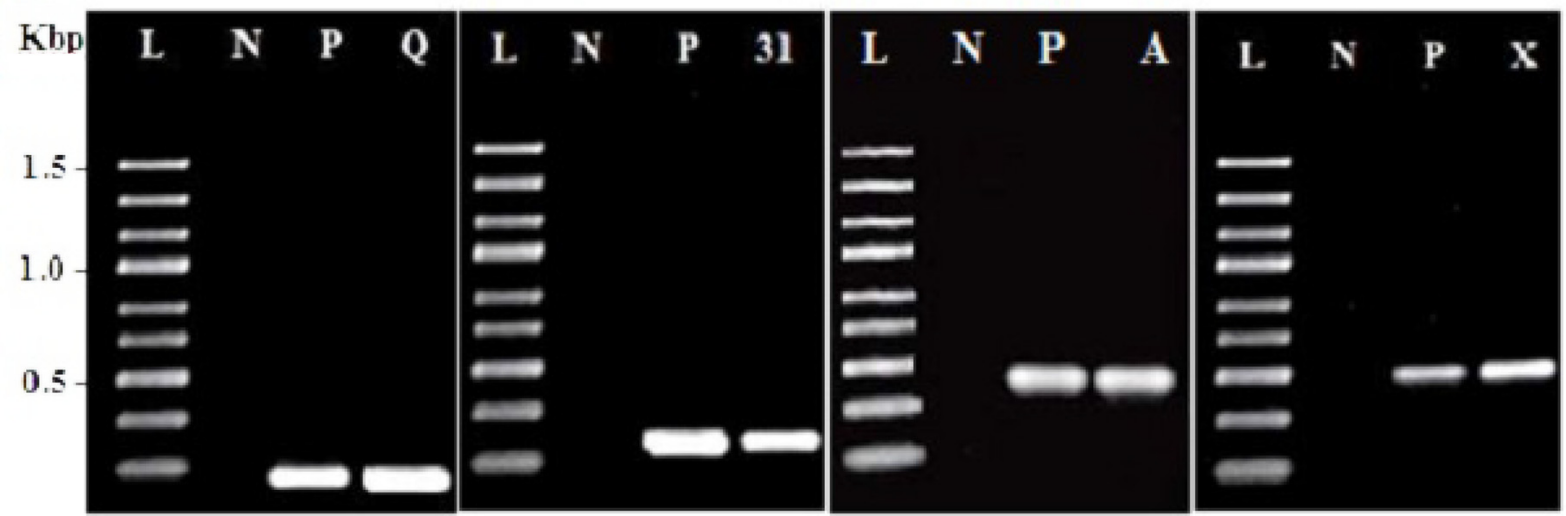
**Amplification results for screening enterocin genes in *Enterococcus faecium* CM33.** L, molecular weight ladder (kbp); N, negative control; P, positive control by using purified DNA from a producer strain; Q, enterocin Q; 31: enterocin 31; A: enterocin A and X: enterocin X.

The production of antimicrobial compounds like bacteriocins, organic acids, and hydrogen peroxide is of the well-designed properties applied to characterize probiotics (Rehaiem et al., 2014). Production of bacteriocins (enterocins) is the useful biotechnological trait of *Enterococcus* bacteria (Franz et al., 2011). The PCR results (**Table 1**; **Figure 1**) showed that *E. faecium* CM33 carried enterocins A, 31, Q, and X structural genes, while the other primers used in this test did not yield any visible band. Franz et al. (2007) classified enterocins A and 31 in the class IIa bacteriocins (pediocin-like bacteriocins). This group of bacteriocins is cationic and shows very effectual anti-*Listeria* activity and permeabilizes the cell membrane to kills target cells. Moreover, results showed that *E. faecium* CM33 has the greatest inhibitory function against *L. monocytogenes*. This fact could be described by the capability of *Enterococcus* bacteria to obtain and replace genetic information by conjugation (Brandão et al., 2010), not only between strains of *Enterococcus* genus, but also with other genera (Strompfová et al., 2008).

The class IIb bacteriocins, two-peptide bacteriocins, also need two diverse peptides (enterocins Q and X) for activity. The production of multiple bacteriocins is not unusual and could be a general aspect of enterococci. Many *Enterococcus* bacteria are able to produce multiple bacteriocins, such as *E. faecium* WHE81 (Ennahar et al., 2001), *E. faecium* KV-B5 (Hu et al., 2010), *E. faecium* NKR-5-3A (Ishibashi et al., 2012), *E. faecium* JCM 5804T (Park et al., 2003), and *E. faecium* DAC2 (Sánchez et al., 2007). Nevertheless, the incidence of numerous enterocin genes in enterococci does not forever link with an advanced bacteriocin activity in their supernatants and not all enterocin genes should be expressed at the same time (Casaus et al., 1997).

*Enterococcus faecium* is the most normally occurring enterococcal species in dairy industry (Morandi et al., 2006) and fermented vegetable (Landeta et al., 2013). This species is also found in raw fruits (Abriouel et al., 2008) and probiotic preparations (Senok, 2009). However the nosocomial infections related to wound, bacteraemia, urinary-tract infections, and abdominal infections are caused by many enterococcal species (Thurlow et al., 2009). *Enterococcus* bacteria, as pathogens, have intrinsic and acquired resistance to many antibiotics that were considered as a major concern. The resistance against antibiotics and the production of virulence factors are two significant factors for the assessment of safety in enterococci. Probiotic strains may harbor antibiotic resistance genes that are able to be transferred to pathogenic bacteria. In particular, the most significant concern among antibiotic resistance is vancomycin resistance since it is the last antibiotics largely efficient against clinical infections by multidrug-resistant pathogens (Klein, 2003). Several studies elucidated the incidence of vancomycin-resistant enterococci in food of animal origin, mainly in *E. faecium* and *E. faecalis* species; although the isolation frequency seems to be lower than in clinical samples (Klein, 2003). In our case, CM33 strain was found to be susceptible to vancomycin, and this lack of resistance associated with the absence of van A, van B and van C_2_ genes. According to the EFSA, if the strain has one or more these genetic elements, this strain is unsafe and could not be applied as a feed additive (Ogier and Serror, 2008). Moreover, no resistance to other antibiotics such as gentamycin, erythromycin, tetracycline, and rifampicin was detected in this colostrum-isolated *Enterococcus*.

The potential pathogenicity of CM33 strain was evaluated by assessing the existence of genes encoding for other virulence factors, as determined by studies in various enterococcal species (Omar et al., 2004). Genes encoding for cell-wall anchored collagen adhesion (*acm*), aggregation substance (*agg*), cytolysin (*cylA*), and gelatinase (*gelE*) were not found in our isolated strain. Besides, the amplification for a sex pheromones gene (*ccf*), which could be involved in initiating conjugation processes (Clewell, 2011) was negative.

One of important properties of strain to be as a potential probiotic is anti-pathogen activity. *In vitro* test under neutralized pH was performed using 13 pathogenic microorganisms and supernatants (metabolites) obtained from the exponential *E. faecium* CM33 culture. The well diffusion technique was performed to evaluate the inhibitory effect of the CM33 strain against 13 pathogenic microorganisms. The *E. faecium* supernatant demonstrated the strong inhibition function against indicator pathogens (**Table 5**). This strain was found to have the strongest inhibition activity against *L. monocytogenes* and *Candida albicans*. It was recommended that *E. faecium* CM33 produced bacteriocins (enterocin A and 31) to inhibit these pathogens.

According to previous studies, the multiple enterocins-producing isolate is likely more capable and could display a wider range of inhibition in inhibiting the growth of undesirable bacteria than a simple bacteriocin producer (Ishibashi et al., 2012). *E. faecium* DAC2 (producer both Ent A and Ent P) produces a higher antagonistic activity than those of the controls *E. faecium* T136 and *E. faecium* P13 (producer of enterocin A and enterocin P, respectively; Sánchez et al., 2007). The production of bacteriocins by LAB helps LAB to be colonized in their habitats and to compete with other bacteria (Garriga et al., 1993). Many factors including decreased pH levels, competition for substrates, and the production of substances such as bacteriocins are caused the antimicrobial activity of LAB (Parente and Ricciardi, 1999).

The competitive inhibition of enteropathogen attachment to epithelial cells by LAB is another important factor. So, the competitive inhibition of adherence of pathogenic bacteria to Caco-2 cells by adhering *E. faecium* CM33 cells was evaluated. This strain was able to strongly inhibit the adhesion of many tested pathogens (**Table 4**). However, the mechanisms by which LAB inhibit pathogen adhesion to human cell lines *in vitro* are not fully understood. Steric hindrance rather than blockage of specific receptors may be involved (Bernet et al., 1993). Additional trials are required to discover the exact mechanism of inhibition observed in this study.

## Conclusion

An identified strain of *E. faecium* from colostrum can present interesting probiotic characteristics. It can stay alive during passing the digestive system and has ability to be colonized on the intestinal epithelial cells. In addition, this strain showed a significant enteropathogen growth inhibiting activity and interference with pathogens adhesion to human colon Caco-2 cells. These features could enable this strain to establish itself in the intestinal tract and to compete with other bacterial species. However, the safety and implications for public health of individual enterococcal strains must be carefully evaluated to fully exploit their industrial potential.

### Ethical Issues

No ethical issues to be promulgated.

## Conflict of Interest Statement

The authors declare that the research was conducted in the absence of any commercial or financial relationships that could be construed as a potential conflict of interest.
